# Vitamin D Status and Behavioral Impulsivity in Anorexia Nervosa: Insights from a Longitudinal Study

**DOI:** 10.3390/nu16152523

**Published:** 2024-08-02

**Authors:** Patrizia Todisco, Alberto De Mico, Paolo Meneguzzo

**Affiliations:** 1 Eating Disorders Unit, Casa di Cura “Villa Margherita”—KOS Group, Via Costacolonna 20, 36057 Arcugnano, Italy; demicoalberto@gmail.com; 2 Department of Neuroscience, University of Padova, Via Giustiniani 2, 35128 Padova, Italy; paolo.meneguzzo@unipd.it; 3 Padova Neuroscience Center, University of Padova, 35128 Padova, Italy

**Keywords:** anorexia nervosa, vitamin D, impulsivity, longitudinal, BART, go/no-go, eating disorders

## Abstract

Anorexia nervosa (AN) is a severe psychiatric disorder marked by extreme weight control behaviors and significant impacts on physical and psychosocial health. This study explores the relationship between vitamin D (Vit-D) levels and impulsivity in women with AN. Forty-six cisgender White women participants were assessed upon admission and before discharge from a specialized eating disorder treatment center, with an average duration of 2.5 ± 0.10 months. Methods included self-reported questionnaires and behavioral tasks to measure impulsivity, alongside serum Vit-D levels. Our results showed significant improvements in Vit-D levels and certain impulsivity measures, such as faster reaction times and fewer errors on the go/no-go task, correlating with higher Vit-D levels. However, no significant correlations were found between Vit-D levels and self-reported impulsivity. These findings suggest that adequate Vit-D levels may enhance cognitive functions related to impulse control in AN. Given this study’s limitations, including its exclusive focus on women and small sample size, future research should involve larger, more diverse populations and randomized clinical trials to better understand the causal relationships and therapeutic potential of Vit-D in managing AN-related impulsivity.

## 1. Introduction

Anorexia nervosa (AN) is a severe psychiatric disorder characterized by dysfunctional eating and weight-control behaviors, an intense fear of gaining weight, and an excessive preoccupation with weight or body shape, significantly affecting self-esteem [1]. This core pathology leads to substantial impairments in physical health and psychosocial functioning due to inadequate energy, protein, and vitamins, profoundly affecting quality of life [2,3].

Vitamin D (Vit-D) deficiency is prevalent in people with eating and weight disorders compared to the general population [4,5,6]. This deficiency may result from dietary restrictions, reduced exposure to the sun due to avoidance behaviors or social withdrawal, and impaired absorption caused by dysfunctional eating patterns [5]. Neurobiological studies suggest that Vit-D receptors in brain regions involved in appetite regulation, mood, and perception of body image may play a role in these disorders [7,8]. Furthermore, Vit-D deficiency has been associated with alterations in neurotransmitter systems such as dopamine and serotonin, which are crucial in the processing of mood and reward in eating disorders (ED) [9].

Impulsivity is a significant factor in the onset and perpetuation of ED, affecting impulse control, emotional regulation, and decision-making skills [10,11]. It encompasses behaviors characterized by rash and unplanned reactions without considering the consequences [12]. Impulsivity manifests differently in various EDs; for example, in AN, it can manifest as sensation seeking and positive urgency [13,14,15], while in disorders characterized by binge eating, it can involve loss of control or compensatory behaviors. In AN, impulsivity has been assessed using various approaches, revealing its potential relevance as a psychological construct [12,14,16]. Impulsivity has been associated with outcomes in AN and other psychological constructs such as obsessive thoughts, showing the complexity of this characteristic [17,18]. However, most of the evaluations were performed using self-reported questionnaires, which have several limitations, despite the existence of various alternative tasks.

Behavioral tasks such as the balloon analog risk task (BART) and the go/no-go task are used to assess impulsivity, highlighting risk-taking tendencies and response inhibition abilities [19]. These tasks provide insights into how impulsivity contributes to maladaptive eating behaviors and the maintenance of ED over time [10,20,21].

The link between impulsivity and Vit-D deficiency has been explored in other conditions such as attention deficit hyperactivity disorder (ADHD), where Vit-D supplementation improved cognitive functions related to impulsivity [22]. However, the literature does not consistently show a direct relationship between Vit-D deficiency and BMI or specific ED diagnoses, underscoring the complexity of the ED etiology [5,23]. Although direct evidence linking Vit-D deficiency and impulsivity in ED is still emerging [24], initial cross-sectional studies suggest that lower Vit-D levels correlate with higher levels of impulsivity in decision-making tasks and a lack of perseverance [24,25].

Therefore, this study aims to longitudinally assess the relationships between Vit-D and impulsivity facets. Our main hypothesis is that Vit-D is linked to different facets of impulsivity, without connections to weight. Our second hypothesis is that changes in plasma Vit-D concentration are linked to changes in impulsive behavior.

## 2. Materials and Methods

### 2.1. Study Design

The present study is a longitudinal evaluation of the relationship between serum 25(OH)D (Vit-D) and different aspects of impulsivity in a group of women with diagnoses of AN. This study was carried out at Casa di Cura ‘Villa Margherita’ (Arcugnano, Vicenza, Italy) in the multidisciplinary specialized ED department. 

Patients were enrolled if they met the following inclusion criteria: (a) a complete diagnosis of AN according to the DSM-5 criteria; (b) no severe medical comorbidity, neurological trauma or disorder, or drug addiction; (c) no drug treatment. Inclusion criteria were evaluated at the beginning of inpatient treatment by a psychiatrist with a structured clinical interview for the diagnosis of DSM-5. Each participant was evaluated at admission (T0) and again before discharge from the ward after an average of three months (T1), which is the maximum duration allowable in the Italian healthcare system.

The Vicenza Ethics Committee approved this study (VI 47/21). This study complies with the provisions of the Declaration of Helsinki. All patients (or parents of patients under the age of 18 years) included in this study agreed to participate and signed an informed consent form.

### 2.2. Instruments

#### 2.2.1. Self-Report Questionnaires

The Eating Disorder Examination Questionnaire (EDE-Q) is a self-reported 28-item questionnaire widely used to assess psychopathological ED [26,27] and validated in Italian populations. The Cronbach α for this study was 0.915. For this study, only the global score was considered. 

The urgency, premeditation (or lack of), perseverance (or lack of), sensation seeking, positive urgency (UPPS-P) impulsive behavior scale is a self-reported 20-item questionnaire used to assess five different domains: negative urgency, positive urgency, lack of perseverance, lack of premeditation, and sensation seeking [28,29]. We applied the validated Italian translation. The Cronbach α for this study was 0.911.

#### 2.2.2. Neuropsychological Evaluation 

Impulsivity was assessed through two different computerized tasks. The first task was the cued go/no-go task, where participants were shown a letter in the center of the screen, either an “X” or an “O”. Participants were instructed to press the corresponding key as quickly as possible only when they saw an X and inhibit their response when they saw an O [30]. Each participant completed four blocks of 30 trials each. This task, a well-established measure of impulsivity, provided various performance metrics: response time (RT) for the first block, mean RT across all blocks, false alarms (FA, pressing the “X” key in response to an “O”), and false negatives (FN, failing to react to an “X”).

The second task was the Balloon Analog Risk Task (BART), a widely recognized task for evaluating risk-taking behavior, decision-making speed, and the ability to wait [31]. In this task, participants saw a balloon on the screen and were required to pump it up to earn money. They could continue pumping to gain 0.05 cents per pump or decide to save their earnings and start with a new balloon. The risk-taking score was calculated as the average number of pumps made in unexploded balloons.

Both tasks were administered in the morning in a quiet room using a 17-inch laptop running OpenSesame software, version 3.0. The order of the participants’ tests was randomized (https://osdoc.cogsci.nl).

#### 2.2.3. Serum Vitamin D

Serum sample levels of total 25(OH)D2 and 25(OH)D3 (Vit-D) were determined by an enzyme immunoassay (UniCel DxI 800 immunoassay system, Beckman Coulter, Inc., Brea, CA, USA) with a sensitivity of 2 ng/mL and intra- and inter-assay coefficients of variation < 10%, considered a valid methodology of analysis. During inpatient treatment, participants received a weekly dose of 8000 IU of cholecalciferol if their serum vitamin D level was insufficient at T0.

### 2.3. Statistical Analysis

First, we assessed the normality of our data distributions. Given that the data did not meet the assumptions of normality, we employed non-parametric statistical methods. To compare repeated measures, we utilized the Wilcoxon signed-rank test. The effect size of significant differences was evaluated using Cohen’s δ. Relationships between different constructs were explored with Spearman’s correlations at both time points. To examine the relationships between changes from T0 to T1, we calculated the difference for each variable by subtracting the baseline value from the T1 value (denoted with the prefix D, for changes in example, DVit-D for vitamin D). The relationships between these changes were then evaluated using Spearman’s rho. To assess the dependency of changes in neuropsychological variables on changes in vitamin D levels, we conducted linear regression analyses. In these models, DVit-D was used as an independent variable to predict changes in various neuropsychological outcomes. All statistical tests were conducted with an alpha level of 0.05 to determine significance. The analyses were performed using SPSS software (version 25.0, IBM Corp., Armonk, NY, USA).

## 3. Results

The sample of this longitudinal evaluation was composed of 46 cisgender White women. The average age was 20.54 ± 2.64 years, and the average time between the two evaluations was 2.5 ± 0.10 months. The demographic and clinical characteristics of the sample at T0 and T1 are reported in Table 1. 

**Figure 1:**
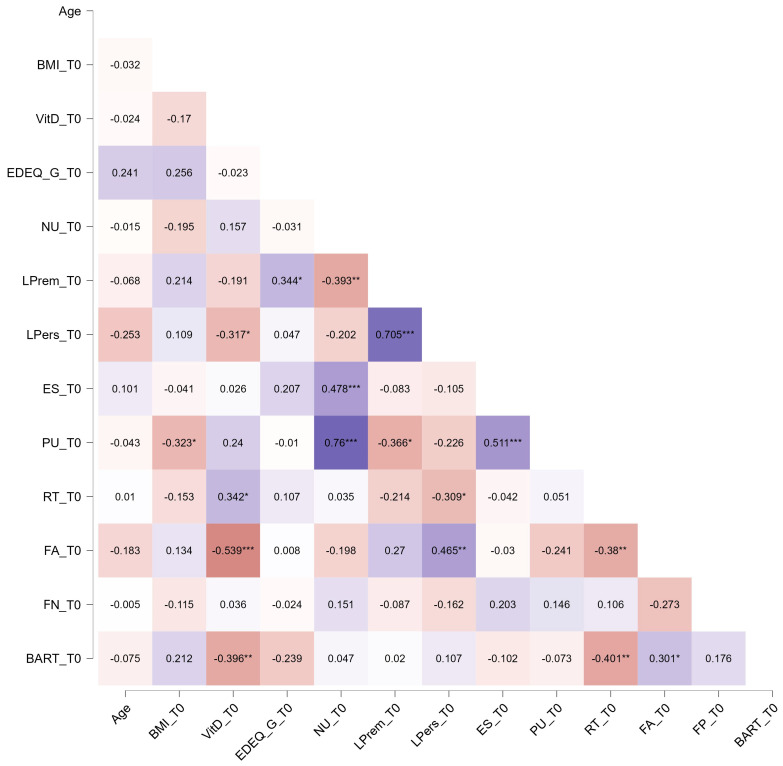
Spearman’s correlations between constructs at T0. Each box reports Spearman’s rho. Purple boxes reported positive correlations, while dark-orange boxes reported negative correlations. Vit-D: vitamin D; NU: negative urgency; LPrem: lack of premeditation; LPers: lack of perseveration; ES: emotional seeking; PU: positive urgency; BART: Balloon Analogue Risk Taking; RT: response time; FA: false alarm; FN: false negative. * *p* < 0.05; ** *p* < 0.01; *** *p* < 0.001.

**Figure 2:**
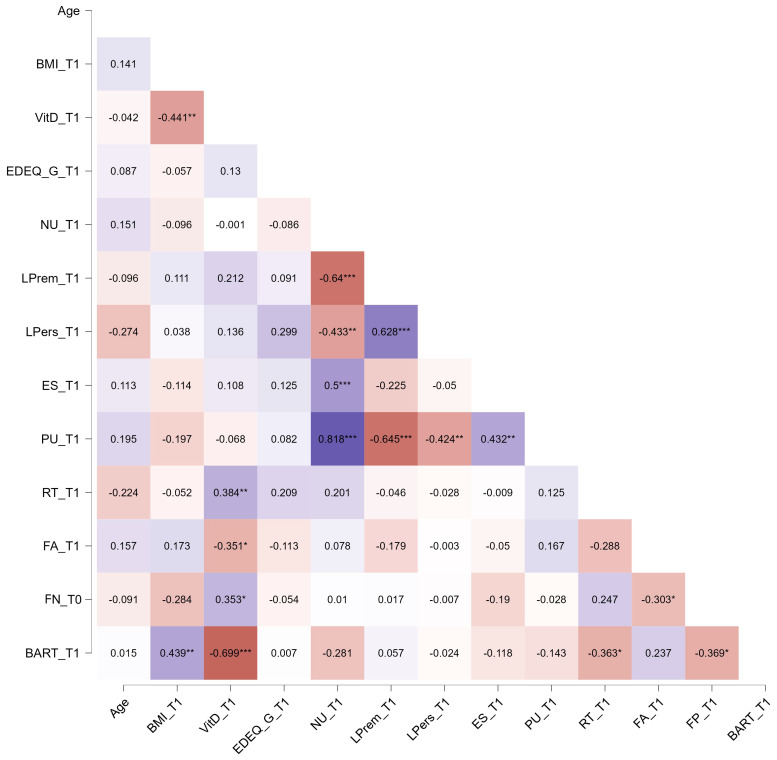
Spearman’s correlations between constructs at T0. Each box reports Spearman’s rho. Purple boxes reported positive correlations, while dark-orange boxes reported negative correlations. Vit-D: vitamin D; NU: negative urgency; LPrem: lack of premeditation; LPers: lack of perseveration; ES: emotional seeking; PU: positive urgency; BART: Balloon Analogue Risk Taking; RT: response time; FA: false alarm; FN: false negative. * *p* < 0.05; ** *p* < 0.01; *** *p* < 0.001.

The correlation analysis showed different relationships between Vit-D and other constructs included in this study (see Figure 1 and Figure 2). In particular, no relationships emerged between Vit-D and psychological evaluations at both times. Various correlations emerged at various time points, indicating distinct relationships between Vit-D levels and task results. For detailed correlations, see Figure 1 and Figure 2. In particular, we identified correlations between tasks at both time points, suggesting convergence in the evaluated constructs. Specifically, there were significant correlations between Vit-D and BART (negative) and RT (positive) at both time points. Furthermore, at T0, Vit-D showed negative correlations with a lack of perseverance and false alarms. In T1, significant correlations were observed between Vit-D and false alarms (negative), false positives (positive), and BMI (negative).

**Figure 3:**
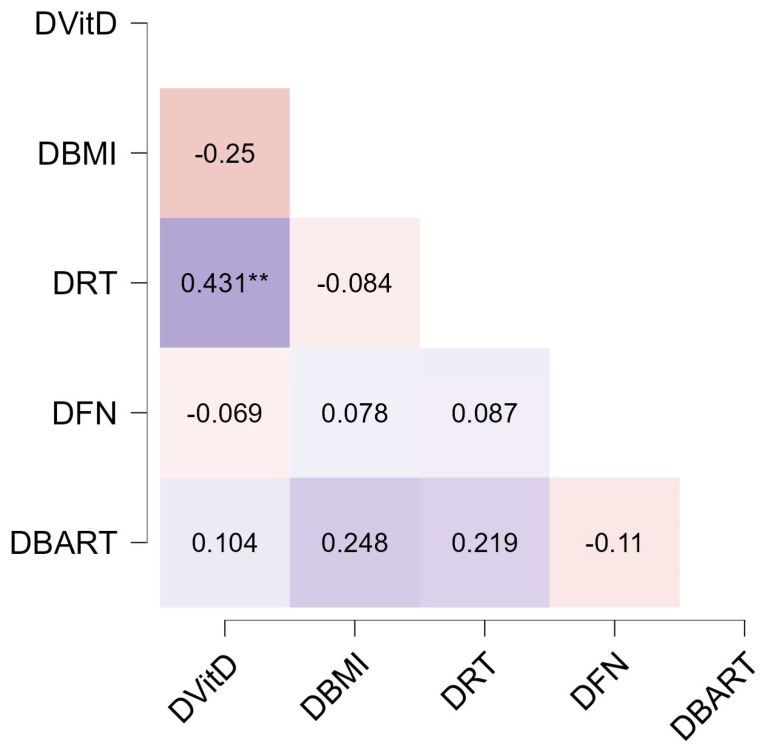
Spearman’s correlations between differences T1-T0. Each box reports Spearman’s rho. Purple boxes reported positive correlations, while dark-orange boxes reported negative correlations. D: differences T1-T0; Vit-D: vitamin D; RT: RT: reaction time; FN: false negative; BART: Balloon Analogue Risk Taking. ** *p* < 0.01.

A significant correlation was found between changes in vitamin D concentration and changes in reaction time (RT) (see Figure 3 for details). No other significant correlations were observed between changes in variables. Furthermore, regression analysis indicated that changes in vitamin D levels can significantly predict changes in RT (F(1,44) = 6.508, *p* = 0.014, R^2^ = 0.13).

Furthermore, when examining the predictive relationship between changes in vitamin D levels in other variables, only FA was found to be significantly predictable (F(1,44) = 4.569, *p* = 0.038, R^2^ = 0.10). No significant predictive relationships were found for the FN scores (F (1,44) = 0.011, *p* = 0.916) or BART (F (1,44) = 1.530, *p* = 0.229).

## 4. Discussion

The present study sought to explore the longitudinal relationships between Vit-D levels and various aspects of impulsivity in women with AN. Our findings provide novel insights into how Vit-D status may influence neuropsychological outcomes in this population, particularly in the context of impulsive behavior.

Specifically, improvements in Vit-D were linked to faster RTs and fewer FAs on the go/no-go task. These findings suggest that adequate Vit-D levels can enhance cognitive functions related to response inhibition and attention, which are crucial in the management of impulsive behaviors. An increase in RTs might indicate a reduction in impulsivity among patients, reflecting improved cognitive control that could extend to everyday life. Impulsivity in AN has been associated with maladaptive cognitive schemas [15], suggesting that future studies could explore different aspects of the evaluated profile. This aspect has already been reported for cognitive performance in the general population [32], calling for different studies. Examining each evaluation point individually, we observed an increase in correlations during the second evaluation, particularly with false negative responses showing increased positive correlations with vitamin D concentration. This finding may suggest the presence of specific impulsive behaviors linked to vitamin D’s neuronal activity, as suggested by pre-clinical studies [33]. However, due to the preliminary nature of this study, further inference is limited.

Our results highlight a complex relationship between Vit-D and impulsivity in individuals with AN. At both time points (T0 and T1), significant correlations were observed between Vit-D levels and specific impulsivity measures such as BART and RT in the go/no-go task. These correlations indicate that higher Vit-D levels are associated with lower risk-taking behaviors and better response inhibition. This aligns with previous research linking Vit-D deficiency with cognitive impairments and impulsive behavior in other populations, such as those with ADHD [22]. Indeed, the implementation of vitamin D has been associated with behavioral changes, potentially due to its anti-inflammatory and neurosteroid hormone effects [34]. From this perspective, more research on the non-bone effects of Vit-D seems to be necessary [35].

However, it is important to note that no significant correlations were found between Vit-D levels and self-reported measures of impulsivity, such as the UPPS-P impulsive behavior scale. This discrepancy underscores the importance of using objective behavioral tasks alongside self-reported questionnaires to gain a comprehensive understanding of impulsivity in EDs. Self-reported measures may be influenced by various biases and may not accurately capture the multifaceted nature of impulsivity. Indeed, impulsivity in anorexia nervosa is a multifaceted aspect, with the co-presence of behavioral and cognitive elements that might require a more robust and thorough evaluation [12,15,18].

Regression analyzes also supported the significant predictive value of changes in Vit-D levels for changes in specific neuropsychological outcomes. Changes in Vit-D levels significantly predicted changes in RT and false alarms, suggesting that improving Vit-D status can lead to better cognitive control and reduced impulsive errors. These findings provide a potential avenue for therapeutic interventions aimed at improving cognitive and behavioral outcomes in individuals with AN through Vit-D supplementation. This has been evaluated in older people, but it might also have an effect on the anorexia nervosa population [36].

The clinical relevance of our findings lies in the potential for Vit-D supplementation to serve as an adjunctive treatment for cognitive and behavioral symptoms in AN. Given the high prevalence of Vit-D deficiency in individuals with eating disorders, the treatment of this deficiency could have broad implications for improving both physical health and psychosocial functioning. Future research should aim to replicate these findings in larger and more diverse samples, including men and individuals with other types of eating disorders. Furthermore, exploring the underlying mechanisms through which Vit-D influences cognitive functions related to impulsivity could provide further insights into targeted interventions. Long-term studies are also needed to determine the sustainability of observed effects and assess the impact of Vit-D supplementation on recovery outcomes in AN. From this perspective, controlling for vitamin D supplementation and the duration of supplementation might help to elucidate the real connections between this hormone and cognitive functions such as impulsivity. In our study, we used a naturalistic approach to supplementation, increasing vitamin D levels in individuals with insufficient concentrations at the beginning of inpatient treatment. However, more rigorous studies are needed.

The potential role of vitamin D supplementation in altering impulsive behaviors has already been evaluated in other psychiatric conditions like ADHD, demonstrating potential positive effects on cognitive functioning, inattention, hyperactivity, and impulsivity [37,38,39]. While the literature on ADHD remains preliminary, data suggest improvements in impulsivity [40]. A proposed explanation considers various psychiatric conditions where serotonin, a neuro-peptide modulated by vitamin D, may play a role [41]. However, eating disorders were not included in this overview. This study has several limitations that should be considered when interpreting its findings. Firstly, the sample is exclusively composed of female cisgender White participants, which limits the generalizability of the results to other ethnicities, males, or nonbinary individuals with AN. Indeed, different cultures might have behaviors that limit sun exposure, with possible effects on this aspect that might require specific evaluations. In addition, the sample size is relatively small, which may affect the robustness and reliability of the findings. The use of self-reported questionnaires has to be considered a potential limitation for the interpretation of the results and might be the reason for the partial results obtained. Replications of this study with larger and more diverse samples are necessary to confirm and extend the results. Furthermore, the observational nature of the study design limits our ability to establish causal relationships between Vit-D levels and impulsivity. Various potential confounders should be considered in the evaluation of disorders, such as the presence of physical activity, which can influence other neuro-peptides like dopamine and serotonin, potentially impacting the changes observed. The use of randomized clinical trials in future research could provide more definitive evidence of the effects of vitamin D supplementation on cognitive functions and impulsivity in people with AN. Conducting RCTs would help us to better understand the underlying mechanisms and validate the therapeutic potential of vitamin D in this context.

## 5. Conclusions

In conclusion, our study provides evidence that improving Vit-D levels through supplementation can positively affect cognitive functions related to impulsivity in women with AN. These findings highlight the potential role of Vit-D as part of a comprehensive treatment approach for AN and, potentially, for EDs. Addressing Vit-D deficiency can help to mitigate impulsive behaviors and improve overall treatment outcomes, thus improving the quality of life of individuals with AN.

## Figures and Tables

**Table 1:** Demographic and clinical characteristics with task responses.

	T0	T1	Z	*p* (δ)
BMI, kg/m^2^	15.70 1.93	18.39 1.99	−5.842	<0.001 1.37
Vit-D, ng/mL	28.57 11.90	44.35 15.31	−4.868	<0.001 1.15
EDE-Q total score	4.31 1.18	2.77 1.17	−4.509	<0.001 1.31
UPPS				
NU	9.51 3.73	9.53 3.97	−1.276	0.202
LPrem	7.80 3.48	7.82 3.40	−0.030	0.976
LPers	8.02 3.60	7.44 3.32	−2.416	0.016 0.17
ES	10.20 3.79	10.89 3.56	−2.343	0.019 0.19
PU	10.58 3.30	11.16 2.98	−1.637	0.102
Go-No go				
RT	326.10 51.54	340.76 43.29	−2.594	0.009 0.31
False alarm	16.52 14.47	7.65 6.39	−3.455	0.001 0.79
False negative	0.65 2.87	0.50 1.68	−0.141	0.888
BART	80.48 17.59	78.07 12.49	−0.699	0.484

The table reports means and standard deviations for each measure. Z represents the Wilcoxon signed rank-test value, and effect sizes are reported for significant changes using Cohen’s δ. Vit-D: vitamin D; NU: negative urgency; LPrem: lack of premeditation; LPers: lack of perseveration; ES: emotional seeking; PU: positive urgency; BART: Balloon Analogue Risk Taking; RT: response time.

## Data Availability

Data supporting the findings of this study are available upon reasonable request from the corresponding author.
